# Echocardiography with Strain Assessment in Psychiatric Diseases: A Narrative Review

**DOI:** 10.3390/diagnostics15030239

**Published:** 2025-01-21

**Authors:** Aleksandra Spyra, Aleksandra Sierpińska, Alexander Suchodolski, Szymon Florek, Mariola Szulik

**Affiliations:** 1 Student Research Group, Faculty of Medical Sciences in Zabrze, Medical University of Silesia in Katowice, 40-055 Katowice, Poland; aleksandra1828@gmail.com (A.S.); sierpinskao@gmail.com (A.S.); 2 Doctoral School, Medical University of Silesia in Katowice, 40-055 Katowice, Poland; alex.suchodolski@gmail.com (A.S.); szymon.florek@sum.edu.pl (S.F.); 3 Department of Cardiology and Electrotherapy, Silesian Center for Heart Diseases, Faculty of Medical Sciences in Zabrze, Medical University of Silesia in Katowice, 40-055 Katowice, Poland; 4 Department of Psychoprophylaxis in Tarnowskie Góry, Faculty of Medical Sciences in Zabrze, Medical University of Silesia in Katowice, 40-055 Katowice, Poland; 5 Collegium Medicum—Faculty of Medicine and Faculty of Applied Sciences, Department of Medical and Health Sciences, WSB University, 41-300 Dąbrowa Górnicza, Poland

**Keywords:** echocardiography, global longitudinal strain, speckle tracking echocardiography, mental disorders

## Abstract

Mental disorders (MDs) are among the major causes of morbidity and mortality worldwide. Individuals with severe MDs have a shorter life expectancy, primarily due to cardiovascular diseases. Echocardiography facilitates the evaluation of alterations in cardiac morphology and function, resulting from various cardiac pathologies. The aim of this review was to explore the current evidence base behind the myocardial deformation observed in echocardiography in patients with MDs. We primarily focused on the data regarding speckle tracking echocardiography. PubMed, using medical subject headings, was searched to identify studies on this topic. The collected data demonstrated changes in myocardial function in schizophrenia, bipolar disorder, depression, anxiety disorder, stressor-related disorder, post-traumatic stress disorder, eating disorders, sleep–wake disorders, substance-related and addictive disorders, neurocognitive disorders, and borderline personality disorder. The recurrent findings included impaired Left Ventricular Ejection Fraction and Left Ventricular Hypertrophy. Global Longitudinal Strain was significantly altered in patients with anorexia nervosa, bipolar disorder, and substance-related disorders. All reported studies support the consideration of cardiology consultations and a multidisciplinary approach in the care of patients with MDs with suspected cardiac dysfunction. Further investigation is warranted to determine the significance and prognostic value of myocardial deformation and strain measurements among individuals with MDs, focusing on the value of early detection, especially in asymptomatic cases.

## 1. Introduction

Mental disorders (MDs) are one of the primary causes of morbidity and mortality worldwide, posing a significant burden on both individuals and society [1]. Individuals diagnosed with MDs, such as schizophrenia and bipolar disorder (BD), exhibit a higher likelihood of developing substance dependence [2]. Referring to the 2022 systematic analysis, the number of patients with MDs in 2019 was estimated at 970 million, with a similar prevalence among men and women [3]. Approximately 20% of Europeans are affected by MD, and the lifetime risk of developing schizophrenia or BD is almost 2% [4]. The two most prevalent MDs are depression and anxiety disorder [3]. MDs typically emerge at an early age and frequently endure a prolonged course, exerting a significant impact on thinking, emotions, behaviour, and social functioning [2,5]. The classification criteria for these disorders are described in the fifth edition of the Diagnostic and Statistical Manual of Mental Disorders (DSM-5) [5]. 

Patients with MDs are at a higher risk of developing various diseases. It is estimated that people with severe MDs have a shorter life expectancy than the general population, and this is related to both mental and physical health [2,6]. The latter is primarily attributed to cardiovascular diseases (CVDs), which are among the primary causes of morbidity and mortality worldwide. Their amalgamation with MDs presents a challenge even for experienced clinicians [7]. 

Echocardiography is widely used to assess cardiac morphology and function, including myocardial deformation resulting from various cardiac pathologies [7,8]. Speckle tracking echocardiography (STE) is a new diagnostic tool that can provide insight into the function of individual myocardial fibre layers. The assessment of global longitudinal strain (GLS) by STE is a sensitive indicator of even subtle myocardial dysfunction. The term “strain” pertains to the degree of contraction of the myocardium. The degree of this deformation can be assessed by the ‘speckle tracking’. ‘Speckles’ are acoustic markers generated by the myocardium, whose movement can be monitored during the cardiac cycle. GLS can be calculated by combining diverse strains from different regions [9]. Previous data suggest that GLS may outperform left ventricular ejection fraction (LVEF) in the early diagnosis and detection of various heart diseases. This is especially evident in individuals with ventricular hypertrophy, in whom GLS, in contrast to LVEF, is reduced at an earlier stage of the disease [9,10].

Due to the escalating incidence of CVDs and MDs, it is imperative to conduct additional research on myocardial deformation in patients with MDs and enhance the prompt detection of changes, especially in asymptomatic cases. In this review, we collected data on myocardial deformation observed in echocardiography in the course of various MDs. We particularly focused on data on GLS evaluated by STE.

### 1.1. Mental Disorders and Their General Impact on the Circulatory System and Heart Function

Among the factors that contribute to increased cardiovascular risk in patients with MDs are chronic inflammation and immune dysfunction (associated with obesity, insulin resistance, type 2 diabetes, acute coronary syndrome, and metabolic syndrome), genetic predisposition, frequent nicotine addiction, sedentary lifestyle, and medication [4].

According to Polcwiartek et al., schizophrenia and BD are correlated with a fivefold elevated risk of cardiovascular and sudden cardiac death, and with an approximately 54% higher risk of coronary artery disease, including myocardial infarction [4]. Cao et al. reported that major depressive disorder, insomnia, and attention deficit hyperactivity disorder (ADHD) may also increase the risk of CVD [11]. Yu et al. reached slightly different conclusions, reporting that anxiety disorder, autism spectrum disorders, and depression are associated with an elevated risk of CVD, while anorexia nervosa, ADHD, BD, obsessive–compulsive disorder, and schizophrenia are not causally related to it [12]. Up to 66% of patients with schizophrenia and BD have undiagnosed CVD before death [4]. Studies showed that it may be related to reduced pain sensitivity [4,13]. The prevalence of atrial fibrillation (AF) is lower in individuals with schizophrenia and BD as compared to the general population. Nonetheless, this may be attributable to an underdiagnosis of AF in these patients [4]. On the contrary, depression has been associated with an elevated likelihood of AF [14]. Chronic inflammation, oxidative stress, and neurohormonal imbalance in patients with MDs are significant factors in the pathophysiology of heart failure (HF), particularly contributing to left ventricular (LV) remodelling and dysfunction. High polygenic risk scores for schizophrenia were correlated with myocardial stiffness and decreased maximum absolute diastolic velocity (early diastolic dysfunction). The reduction in LVEF and diastolic dysfunction may be a direct consequence of the antipsychotic treatment, or it may be associated with early diffuse myocardial fibrosis and subclinical myocarditis, contributing to the development of concentric LV remodelling. Additionally, there are reports indicating that the incidence of mitral and aortic valve diseases in patients with schizophrenia and BD is higher than in the general population [4].

Antipsychotics have a detrimental impact on cardiovascular risk; however, they are advantageous in terms of patient mortality, primarily due to enhanced psychiatric stability and compliance with medical interventions. It is noteworthy that antipsychotic treatment is associated with corrected QT prolongation and ventricular arrhythmias, specifically torsade de pointes, thereby elevating the likelihood of sudden cardiac death. Several antipsychotic medications have been linked to myocarditis [15,16]. There is also a correlation between antipsychotic medication and reduced LVEF in patients with schizophrenia, treated with clozapine [4]. The exact mechanism of clozapine’s cardiotoxicity is unclear. Myocardial damage is thought to be related to both direct effects of the drug on cardiomyocytes and indirect effects through modulation of the immune system response [14]. On the other hand, there have been reports of an elevated risk of CVD among patients taking antidepressants [11].

### 1.2. Speckle Tracking Echocardiography (STE) and Global Longitudinal Strain (GLS)

STE is a novel approach to the evaluation of ventricular function [17]. Myocardial strain is a percentage change in myocardial fibre length relative to its initial length. This term describes the different spatial components of cardiac contraction, both globally and regionally. Strain rate, a temporal derivative of strain, represents the time interval over which deformation occurs. Imaging myocardial deformation can be performed with echocardiography using tissue Doppler imaging (TDI) or STE [18]. However, STE has several advantages over TDI, including angle independence, enhanced repeatability, and expeditious image acquisition [9].

The strain assessment can be conducted in the longitudinal, circumferential, and radial planes [18]. Strain and strain rate may be evaluated in individual myocardial segments; however, in cardiology, global values have important prognostic and therapeutic implications: GLS, global circumferential strain (GCS), and global radial strain (GRS) [17,18]. The reference ranges for LV GLS and right ventricular (RV) free-wall strain are between −24% and −16% and −35% to −17%, respectively. Higher GLS and GCS (more negative values) and higher GRS (more positive values) indicate a more pronounced contraction in the respective dimension. A less negative value generally reflects impaired GLS. Left atrial (LA) and right atrial reservoir strains are 17% to 49% and 17% to 59% [18,19]. The value range can be explained by the difference between software and vendor variability, but particularly by sex and age characteristics [9].

The assessment of GLS using STE provides a more precise evaluation of systolic function and facilitates earlier detection of subclinical LV dysfunction than LVEF. Longitudinally oriented myocardial fibres are more susceptible to ischemia-related damage due to their primarily subendocardial localization [18,20]. The subendocardial damage resulting from this process leads to a decrease in the regional longitudinal strain within the infarct region [20]. Moreover, numerous studies have highlighted the role of STE in the diagnosis and treatment of cardiomyopathy, valvular heart disease, left ventricular hypertrophy (LVH), and HF with both reduced and preserved LVEF [9,18]. Hence, longitudinal strain analysis can be employed to detect subclinical modifications in cardiac systolic function in patients, including those with MDs.

In a 2023 study, Karakayali et al. found that GLS values were less negative in patients with BD and schizophrenia compared to healthy individuals. Moreover, there were no significant differences in GLS between patients with these two diseases [10]. Similarly to the STE discussed above, velocity vector imaging (VVI) can be used to assess LV deformation. VVI analyzes the velocity vector of myocardial tissue, as it quantifies the velocity and direction of myocardial motion at multiple points in the LV, based on a combination of mitral annular motion, tissue–blood border detection, and speckle tracking [18].

The majority of blood fills the LV physiologically during the passive phase of early diastolic filling. As a result, the early diastolic transmitral flow velocity (E wave, E) is usually greater than the late diastolic transmitral flow velocity (A wave, A). When LV wall stiffness is present, early diastolic passive ventricular filling is impaired, and the A wave of the mitral valve is larger than the E wave [8].

## 2. Materials and Methods

A comprehensive literature search was conducted using the PubMed database. The search was performed from May 2024 to July 2024. To find relevant data concerning our topic, we chose to use the following MeSH Terms/Title: “echocardiography”, “dementia”, “Alzheimer’s disease”, “delirium”, “hallucinosis”, “catatonia”, “schizophrenia”, “bipolar disorder”, “depression”, “anxiety disorder”, “mild cognitive impairment”, “mental disorder”, “personality disorder”, “addiction”, “schizoaffective disorder”, “stress-related disorder”, “obsessive–compulsive disorder”, “post-traumatic stress disorder”, “anorexia nervosa”, “bulimia”, and “insomnia”. We considered a wide range of studies. The data obtained are discussed in the next paragraphs.

## 3. Results

### 3.1. Schizophrenia Spectrum and Other Psychotic Disorders

It is estimated that about 1% of the population suffers from schizophrenia. This condition frequently affects young adults, and its manifestations include hallucinations and delusions, as well as disorganized behaviour. The development of schizophrenia is influenced by genetic, environmental, and neurobiological factors [21]. The schizophrenia spectrum includes disorders such as schizophrenia, schizoaffective disorder, and schizophreniform disorder [22]. There are studies in the literature describing changes in echocardiographic examination in patients with schizophrenia and schizoaffective disorders (Table 1).

A 2016 study of 40 patients with schizophrenia was carried out by Korkmaz et al. The findings of this study indicate that patients with schizophrenia had worse LV systolic and diastolic functions. The Tei Index (an index that assesses both LV systolic and diastolic function) was 0.61 ± 0.19 in the patient group, and 0.39 ± 0.10 in the control group. LVEF was 58% ± 5 and 62% ± 3, respectively. There were differences in isovolumic relaxation time (IVRT) (indicator of diastolic dysfunction) and TDI early diastolic mitral annular velocity (e′) and e′/a′ (e′/late diastolic mitral annular velocity) ratio (indicator of diastolic early-stage dysfunction) among the groups [23]. In a 2023 study, Karakayali et al. recruited 47 patients diagnosed with BD and 44 patients with schizophrenia. There was no difference in GLS values between these two groups. Moreover, both groups’ GLS values were significantly less negative than in the control group [10].

Searching for the cause of cardiac dysfunction in patients with schizophrenia, we found two studies indicating an association between cardiac dysfunction and antipsychotic drugs [29,30]. According to Chow et al., patients with schizophrenia taking clozapine showed impaired LVEF, less negative GLS and reduced early diastolic strain rate (index suggesting diastolic dysfunction). The greatest difference in GLS was noted between patients with schizophrenia treated with clozapine and controls. Compared with healthy individuals, patients with schizophrenia, both taking and not taking clozapine, had significantly higher Tei index [29]. A 2019 cross-sectional study included 64 patients taking long-acting injectable (LAI) medications (olanzapine vs. risperidone). Among patients with a longer duration of antipsychotic therapy, E and A waves and GLS measurements significantly correlated with patients’ myocardial contractility. Patients with prolonged pre-LAI treatment periods had significantly less negative GLS, reduced E and A waves, increased E/A ratio and IVRT. Olanzapine-treated patients showed significantly better myocardial contractility, higher E wave, more negative GLS, and more positive GRS values compared to risperidone-treated patients. Most patients had abnormal LA volume index, and LA dimensions did not change after the treatment. There was no difference in LVEF between groups [30]. Similarly, in a case report of a patient with schizoaffective disorder, Mizera et al. described dilated LV with highly diffusely reduced systolic LV function (LVEF = 15%) due to treatment with clozapine and lithium. LA was also dilated in the parasternal long axis at 54 mm (normal range < 40 mm). Visually, there was impaired RV function. The mitral valve appeared to be slightly insufficient [31].

### 3.2. Bipolar and Related Disorders

BD is characterized by the occurrence of severe mood disturbances [32]. Among the bipolar and related disorders listed in the DSM-5 classification, we found studies on bipolar I disorder (BD-I) and bipolar II disorder [33]. In the course of BD-I, there are episodes of depression and mania. This type usually has an acute onset. Bipolar II disorder has a more chronic course, during which episodes of depression often occur. Episodes of hypomania are also observed [34]. The incidence of BD-I is about 1% [32]. During an episode of mania/hypomania, the patient experiences an improved mood and increased energy. Episodes of depression are characterized by low mood and lack of pleasure [35].

Based on our literature search, in addition to the study by Karakayali et al. [10], we found four studies reporting echocardiographic findings in patients with BD (Table 1) [7,8,24,25]. In the cross-sectional study conducted by Chen et al., patients with BD had significantly greater LV relative wall thickness (RWT), lower E/A ratio (*p* = 0.023), and less negative GLS (*p* = 0.047) compared to the control group [24]. According to a 2022 cross-sectional study, BD-I patients had significantly higher mean values of interventricular septum (IVS) and LV end-diastolic diameter (LVEDD) than the control group. Moreover, patients with BD-I had significantly lower E/A ratio and less negative GLS; however, the result for GLS was on the verge of statistical significance (*p* = 0.029). Multiple linear regression showed that body mass index was negatively correlated with E/A ratio in BD-I patients [8]. A cross-sectional study conducted by Tsai et al. showed that patients with BD had significantly higher rates of LVH, higher mean values of LV mass index (LVMI), and LVEF [25]. Duz et al. assessed the myocardial strain in patients with BD depending on the phase of the disease. Patients in the manic phase had the most negative GLS. Less negative values were noted in patients in the euthymic and depressive phases, and the least negative in the control group. Additionally, patients in mania had the highest E/A ratio [7]. Chen et al. evaluated whether lithium treatment was associated with favourable LV geometric patterns in patients with BD. The results of this study showed that patients who received lithium had lower RWT and less LV concentricity than the patients in the group without lithium therapy [36].

### 3.3. Depressive Disorders

Depression is the primary contributor to the burden of mental health-related diseases, affecting approximately 280 million individuals and resulting in more than 47 million disability-adjusted life-years in 2019. Depression is also correlated with premature mortality due to other illnesses and suicide [37]. The symptoms differ in duration, the timing of disease onset or presumed etiology [38]. Depression may be a distinct risk factor for the development of CVDs [27]. Studies examining the impact of depression on the structure and function of the heart muscle are included in Table 1.

In an observational study, Shi et al. found that depression was a significant risk factor for LVH in hypertensive patients, particularly in those younger than 60 years of age, males, and those with higher education. In this study, patients were considered to have hypertension if their systolic blood pressure was more significant than 140 mmHg, diastolic blood pressure was greater than 90 mmHg, or they had taken antihypertensive medication within the previous 2 weeks. Individuals with hypertension and depression had a higher risk of LVH than those with hypertension alone. Linear regression analysis showed a positive effect of depression intensity in the Hamilton Depression Rating Scale (HAM-D) on LVMI. Additionally, patients with HAM-D scores ≥ 20 took more antihypertensive drugs compared to other examined respondents. The results of this study indicate the need to control blood pressure in patients with depression [26]. According to the study by Kim et al., individuals with moderate to severe depression showed subclinical changes in the structure and function of the LV. The parameters of LV diastolic function, such as the A wave and E/A ratio, e′ and E/e′ ratio, were progressively worse in the order of mild, moderate, and severe depression. Individuals with moderate to severe depression showed significantly higher LVMI (*p* = 0.019) and lower e′ (*p* = 0.006), compared with individuals without depression. Additionally, in linear regression models, the presence of depression was independently associated with lower e′ [27]. A cross-sectional study by Santiago et al. reported that clinically significant depressive symptoms were associated with reduced e′ [28].

### 3.4. Anxiety Disorders

Anxiety disorders are conditions that are correlated with either generalized or situational responses to perceived threats. They constitute one of the most prevalent mental health issues. It is estimated that 4.05% of the world’s population has anxiety disorders, which is about 301 million people. Anxiety disorders typically manifest early in life and endure a persistent progression, resulting in a significant functional decline. For some people, it is associated with specific environmental stimuli, which can lead to phobias. Others may encounter severe episodic stress, such as in the case of panic disorders. If untreated, persistent anxiety can result in a multitude of other health issues, including hypertension, CVD, and dementia [39]. The studies related to anxiety disorder are presented in Table 2. 

Kong et al. found that LVMI was higher in patients with anxiety disorder and hypertension than in hypertension alone [40]. Similar conclusions were reached by Zhu et al. [41]. Baykiz et al. conducted a prospective observational study to investigate the association between mental health disorders and subclinical early myocardial contractile dysfunction on LV GLS imaging in patients who recovered from COVID-19. In total, 45.0% of COVID-19 patients had some degree of anxiety after recovery. GLS values were less negative in the study groups compared to the control group. There was a significant negative correlation between GLS and the Depression, Anxiety, and Stress Scale-21 (DASS-21) total score, the DASS-21 anxiety subscale score, the Impact of Events Scale (IES-R) total score, as well as the IES-R intrusion subscale score. The DASS-21 total score was found to be an independent predictor of GLS values. Patients who recovered in the hospital had a lower E/A ratio compared to the home recovery group and the control group. This subgroup of respondents also had a larger LA diameter, but this difference was on the verge of statistical significance (*p* = 0.045) [42]. In a prospective study, Oksuz et al. showed that inter- and right intra-atrial electromechanical delay (AEMD) measured by TDI was significantly prolonged in patients with anxiety disorders compared with the control group. The precise mechanism behind the prolongation of AEMD remains uncertain. It has been suggested that dysregulation of the sympathetic nervous system in patients with anxiety disorders may be a potential cause for the prolongation of AEMD. It is noteworthy that a prolonged intra- and interatrial delay serves as a precursor to the onset of AF [43].

### 3.5. Trauma- and Stressor-Related Disorders

Stress is caused by physiological responses to negative and upsetting stimuli. One or more traumatic events may result in acute stress disorder. The most prevalent symptoms include distress, sleep issues, negativity, avoiding stress-related factors, irritability, difficulty concentrating, guilt, anxiety, and panic [56]. Trauma- and stressor-related disorders listed in the DSM-5 include conditions such as post-traumatic stress disorder (PTSD), acute stress disorder, adjustment disorders, and other specified trauma- and stressor-related disorders [57]. PTSD may arise subsequent to astonishing, alarming, or perilous occurrences, such as car accidents, natural disasters, sexual assault, violence, or military combat [56]. It appears that PTSD is relatively rare compared to other disorders, with prevalence rates of around 1–2% in the population [58].

In the study by Kim et al., GLS was significantly less negative in patients with higher global stress severity index (GSI) (values ≥ 50) compared to patients with GSI < 50. GSI showed significant correlations with GLS. The value of GLS correlated with paranoid ideation, phobic anxiety, anxiety, compulsivity, depression, and interpersonal sensitivity. GLS did not seem to be associated with somatization, hostility, or psychoticism [44]. A 2019 cross-sectional study found that women with PTSD showed a greater E/e′ ratio and lower velocity of propagation. These findings suggest that women with PTSD exhibit decreased LV diastolic function, which may partially contribute to the elevated risk of CVD in later life [45] (Table 2). 

### 3.6. Eating Disorders

In the course of eating disorders, in addition to often drastic weight loss leading to impaired body functioning and increased mortality, impaired psychosocial functioning is observed. The causes of these disorders include biological, psychological, and sociocultural factors. Anorexia nervosa and bulimia can affect people of all ages and both sexes, but they are especially frequent among young women, with the prevalence estimated at 0.3% and 1%, respectively [59].

There are many reports in the literature on the influence of eating disorders on heart function (mainly anorexia nervosa), regarding LVEF, LV mass (LVM), LVMI, LV and LA dimensions, TAPSE, mitral valve prolapse, and the presence of pericardial effusion [46,47,48,49,50,51,52,60,61,62,63,64,65,66,67,68,69,70]. In our review, we predominantly included studies that focused on myocardial deformation assessed by STE (Table 2).

According to Krantz et al., patients with anorexia nervosa had reduced LVEDD and LVM compared with healthy controls. There was pronounced heterogeneity in segmental longitudinal strain between the cohorts. Patients with anorexia nervosa showed less negative basal strain relative to the control cohort but more negative apical strain. There was no significant difference in the mid-segment between the patients and control groups. Among patients with anorexia nervosa, a longer duration of eating disorder was significantly associated with less negative GLS. Additionally, a significant interaction between lower LVM and less negative basal strain was observed (beta—0.15, SE  =  0.07, *p*  =  0.003). In contrast, LVM was not correlated with the duration of the eating disorder [46]. In the prospective study by Justine et al., patients with anorexia nervosa had more negative GLS compared with controls. Due to more negative GLS and lower blood pressure, the area under the pressure-strain loop (representing global myocardial work) was shifted leftward and downward. No changes in circumferential strain were observed [47]. Galetta et al. found that patients with anorexia nervosa had reduced LVM with normal systolic function and diastolic patterns, characterized by lower A, comparable E, and greater E/A ratio than constitutionally thin group and controls [48]. Sheggi et al. demonstrated that patients with anorexia nervosa had a greater prevalence of pericardial effusion, reduced LVM, and less negative values of GLS and basal longitudinal strain. The bull’s eye mapping showed a plot pattern with blue basal areas in 18 of 48 patients with anorexia nervosa versus 1 of 44 controls [49]. Borgia et al. examined 38 children with anorexia nervosa. Patients with anorexia nervosa cardiopathy had less negative GLS, reduced LVEDD and LVM, higher E values, E/A and E/e′ ratios, and LV sphericity index compared to the control group and children with anorexia nervosa without cardiopathy. As a result of treatment of the underlying disease, GLS improved, whereas LVEDD, E/A ratio, and sphericity index remained unchanged [50]. Hanachi et al. studied 124 patients with restrictive and binge/purging anorexia nervosa. Impaired LVEF was found in 15% of patients, with a predominance in individuals with binge/purging anorexia nervosa. Patients had significantly reduced LVM, LVEDD, myocardial systolic and diastolic LV septal and lateral, and RV velocities compared to the control group. Additionally, maximum LV diastolic velocity values were significantly associated with hypertransaminasemia and tended to be associated with low whole-body fat-free mass index [51]. Another study by Galetta et al. showed that patients with anorexia nervosa had lower LVM, lower peak systolic velocity at myocardial segments (s′) of both lateral wall and septum, and comparable e′, a′, and e′/a′ ratio. The E/e′ ratio was significantly greater in anorexic patients than in the control group. In the anorexia nervosa group, s′ was significantly related to LVMI, at both septum and lateral wall levels. Based on these studies, it is possible to conclude that anorexia nervosa causes LV systolic dysfunction associated with reduced LVM. TDI may provide valuable information in identifying regional LV dysfunction in this particular group of patients [52].

### 3.7. Sleep–Wake Disorders

Sleep disorders are a group of conditions that affect standard sleep patterns. They are a common clinical problem, affecting nearly a third of adults. These conditions can hurt overall health, safety, and quality of life [71]. Diseases classified as sleep–wake disorders include, among others, insomnia and circadian rhythm sleep–wake disorders [72]. The studies regarding sleep–wake disorders are presented in Table 2. 

Strand et al. showed no evidence of an association between increasing insomnia symptoms and any outcome variable related to LV function. Similarly, there was no evidence of an association between individual insomnia symptoms (difficulty falling asleep, repeated awakenings, and early awakenings) and any measure of LV function [53]. Gump et al. examined 176 children aged 9–11 years. Poor sleep quality was associated with significantly greater LVM. RWT and RV dimensions were also significantly associated with sleep characteristics [54]. According to Lee et al., short sleep duration (<7 h) was associated with greater LVH and RWT. No significant association was observed between sleep and LV diastolic function in this study. Poor sleep quality was not correlated with adverse cardiac outcomes in individuals with standard sleep duration [55].

### 3.8. Substance-Related and Addictive Disorders

The prevalence of substance use disorders (SUDs) is high, and it varies depending on the country, type of drug used (highest for tobacco and alcohol use disorders), and demographic and socioeconomic characteristics of the population. SUD rates are higher in men and younger people. Tobacco and alcohol use are the second and seventh risk factors associated with premature death. Addiction leads to the exacerbation of many acute and chronic diseases, including CVDs [73]. 

Maceira et al., using cardiovascular magnetic resonance imaging with feature tracking analysis, showed significant differences in GLS, GRS, GCS, and circumferential dyssynchrony index between asymptomatic cocaine-dependent drug users with mildly decreased LVEF and preserved LVEF, as well as healthy controls. Cocaine addicts with mildly decreased LVEF had the least negative GLS and GCS, and the least positive GRS, compared to the other groups. Moreover, cocaine-dependent individuals with preserved LVEF had significantly less negative GLS and GCS values and less positive GRS values compared to the control group [74].

In Table 3, we presented literature data on echocardiographic changes in patients with SUDs. We analyzed the results of five studies on alcohol dependence [75,76,77,78,79] and two on methamphetamine, heroin and synthetic cannabinoids (SCB) [80,81]. 

Kucuk et al. showed that LV GLS and GCS were significantly less negative in alcoholics compared to controls. No difference in GRS was observed. Additionally, patients in the alcoholic group had a larger diameter of LA [75]. Wang et al. investigated how the amount and timing of alcohol consumption affect LV dysfunction. They divided patients with chronic alcoholism into mild, moderate, and severe drinking groups. The moderate drinking group had lower LV diameter and wall thickness than the mild drinking group and controls. The severe drinking group had dilated LV diameter, thickened LV walls, and decreased LVEF and E/A ratio compared to other groups and controls. Generally, the moderate and severe drinking groups demonstrated less negative GLS and GCS, and less positive GRS values compared with the mild drinking group and the control group. In addition, the severe drinking group had lower mitral annular and apex rotation angles than other groups [76]. Kocabay et al. found that LA function was reduced in chronic alcohol abusers [77]. Another study showed that patients with alcoholism had larger LA volumes (max, min, and pre-atrial contraction volume). GLS was less negative in moderate and severe drinking groups, compared with the mild drinking group and healthy individuals. The least negative values were observed in the severe drinking group. E/A ratio showed a progressive decrease according to the grades of alcoholism [78]. According to Meng et al., patients with the highest alcohol consumption had significantly higher RV end-diastolic dimension, lower TAPSE, and less negative GLS compared to patients with lower alcohol consumption and the control group [79]. Zhang et al. examined 53 asymptomatic methamphetamine abusers and demonstrated that global area strain (GAS), GLS and GCS were remarkably less negative in methamphetamine abusers compared to the control group. However, LV End-Systolic Volume, LV End-Diastolic Volume, LVEF, E/A, E/e′ ratios, and GRS did not differ significantly. The areas under the receiver operating characteristic curve for GAS were the greatest among all global LV strains derived from 3D STE (GAS vs. GLS, GCS, and GRS, 0.95 vs. 0.76, 0.69, and 0.61, respectively). A cutoff value of −30.3% of GAS had a sensitivity of 91.8%, specificity of 91.6%, and accuracy of 91.3% to distinguish methamphetamine abusers from healthy controls, indicating a role for GAS in screening and detecting subclinical LV dysfunction. According to this study, myocardial abnormalities can be detected by 3D STE in asymptomatic methamphetamine abusers [80]. Demirkıran et al. investigated cardiac structure and function using echocardiographic strain imaging in heroin and SCB users. SCB users had less negative GLS, apical 2-chamber, and apical 4-chamber longitudinal strain, indicating that SCB may be a potential cause of subclinical LV dysfunction. In the case of heroin users, all three mentioned values were comparable to controls, suggesting that heroin has no impact on LV function [81].

### 3.9. Neurocognitive Disorders

Dementia is a general decline in memory and other cognitive skills severe enough to limit a person’s ability to perform daily activities. The most common include Alzheimer’s disease (AD), Lewy body dementia and frontotemporal degeneration. AD is the most common cause of dementia, accounting for approximately 70% to 80% of all cases [88]. AD and related dementias have a high prevalence, affecting more than 10% of those aged 65 years or older [89]. The studies analyzed are included in Table 3.

Sanna et al. showed that the prevalence of diastolic dysfunction was twice as high in patients with AD compared to the control group. Patients with AD had greater maximum wall and IVS thickness, significantly lower E/A ratios and a trend toward a higher E/e′ ratio than the control group [82]. Nowak et al. demonstrated that among survivors of acute HF decompensation, patients with subsequent positive screening diagnosis of dementia (SDD) were characterized by a thicker IVS and LV posterior wall, higher RWT, and higher aortic valve peak gradient as compared to subjects without SDD. Higher RWT reflecting LV geometry changes, but not hypertrophy, was independently but moderately associated with a greater likelihood of SDD [83]. Hashemi et al. evaluated cognitive function in 85 patients who underwent coronary artery angiography. Lower LVEF was associated with lower scores on visuospatial function, executive function, processing speed/attention, and verbal memory capacity. The correlation between lower executive function and processing speed/attention and a larger LA size was also observed. The e′ index was associated with a lower executive function capacity in patients with cognitive impairment, but this difference was on the verge of statistical significance (*p* = 0.05) [84]. Çalık et al. examined 29 patients with AD. They had lower E and higher A waves, deceleration time of peak E velocity, and isovolumetric relaxation time. E′ was significantly lower in AD patients; however, a′ and E/e′ ratios were similar to the control group. AD patients had higher stiffness index and pressure-strain elastic modulus, lower distensibility, and longer inter-AEMD compared with the controls [85].

### 3.10. Personality Disorders

Personality disorders are widespread, maladaptive, and persistent patterns of behaviour, cognition, and mood. The prevalence of personality disorders is 6.1% [90]. In our review, we found two studies relating to borderline personality disorder (BPD) (Table 3) [86,87]. Engemann et al. examined 50 females diagnosed with BPD. Somatically healthy women with BPD had less negative GLS values compared to the control group. There was a correlation between childhood trauma and GLS; however, it did not reach the statistical significance threshold [86]. According to Aweimer et al., patients with BPD had significantly less negative GLS, thicker septum and lower TAPSE compared to the control group [87].

## 4. Conclusions

In conclusion, our review highlights the fact that echocardiographic abnormalities are detected more frequently in patients with MDs compared to the general population. In schizophrenia, BD, anorexia nervosa, anxiety, stress-related and addictive disorders, and BPD, the main findings are less negative GLS values. Depression is mostly related to higher values of LVM, A, a′ and E/e′ ratio, as well as lower e′ and E/A ratio. In neurodegenerative diseases, frequently recurring changes include greater maximum wall and IVS thickness, lower E/A and higher E/e′ ratios. Echocardiography is used to evaluate alterations in cardiac morphology and function, resulting from various cardiac pathologies. GLS is a valuable prognostic factor that can detect subclinical LV dysfunction before LVEF decline. An early detection of myocardial dysfunction is crucial to identify patients with MD at higher risk of CVD and warrants further research into possible preventive strategies.

## 5. Future Directions

Individuals with MDs exhibit a shorter life expectancy, primarily due to CVDs. The studies mentioned in this review substantiate the necessity for cardiology consultations and a multidisciplinary approach in treating patients with MDs. There is undoubtedly a requirement for guidelines concerning cardiac health in psychiatric patients, as it would serve as a valuable instrument for determining the extent of cardiac care required. Further studies are needed to evaluate pathological changes in the structure and function of the myocardium in patients with MDs.

## Figures and Tables

**Table 1:** Echocardiographic findings in patients with schizophrenia, bipolar disorder, and depression.

Study	Psychiatric Disease	Study Group Characteristics	Echocardiographic Findings
Korkmaz et al. [23]	Schizophrenia	Patient group N: 40 Age: 38.5 ± 12.8 Control group N: 40 Age: 35.7 ± 9.8 Both sexes	- Echocardiographic myocardial performance, LV systolic, and diastolic functions were found to be worse in patients with schizophrenia compared to the control group
Karakayali et al. [10]	Schizophrenia, Bipolar Disorder	Patients with schizophrenia group N: 44 Age: 42 ± 10 Patients with BD group N: 47 Age: 44 ± 12 Control group N: 52 Age: 42 ± 10 Both sexes	- There was no significant difference between the groups in LVEF, LVEDD, LVESD, IVSD, PWD, and E/A ratio - GLS values were less negative in BD and schizophrenia patients compared to controls (−16.1 ± 1.6 vs. −15.9 ± 1.5 vs. −21.7 ± 1.6)
Chen PH et al. [24]	Bipolar Disorder	Patient group N: 52 Age: 44.8 ± 12.4 Control group N: 52 Age: 44.8 ± 12.2 Both sexes	- Patients with BD had a significantly higher LVMI, greater LV RWT, lower E/A ratio, and less negative GLS compared to the control group
Chen PH et al. [8]	Bipolar Disorder	Patient group N: 48 Age: 57 ± 7.6 Control group N: 31 Age: 56.5 ± 5.6 Both sexes	- Patients with BD-I had significantly higher mean values of IVSD and LVEDD compared to controls - BD-I patients exhibited significantly lower E/A ratio and less negative GLS - BMI was negatively correlated with E/A ratio in patients with BD-I
Tsai et al. [25]	Bipolar Disorder	Patient group N: 60 Age: 38.7 ± 11.4 Control group N: 50 Age: 36.6 ± 8.7 Both sexes	- Patients with BD had significantly greater LVMI, rate of LVH and LVEF compared to controls
Duz et al. [7]	Bipolar Disorder	Patient group N: 150 50 in depressive phase, 50 in manic phase, 50 in euthymic phase Age: 17–65 Control group N: 50 Age: 18–65 Both sexes	- E/A ratio was the highest in the manic phase of BD - The manic phase group exhibited the most negative GLS, followed by the euthymic, then depressive phase group, and then healthy controls (−21.51 vs. −20.75 vs. −20.25 vs. −19.0)
Shi et al. [26]	Depression	Patient group N: 353 Age: 59.29 ± 9.87 Both sexes	- Depression was a significant risk factor for LVH in hypertensive patients, particularly in those younger than 60 years of age, males, and with higher education - Linear regression analysis showed a positive association between HAM-D and LVMI
Kim et al. [27]	Depression	Patient group N: 2420 1732 participants without depression, 547 had mild depression, 141 had moderate to severe depression Age: 40–79 Both sexes	- Patients with moderate to severe depression had a higher A, a′, E/e′ ratio, and LVM - Patients with moderate to severe depression had a lower E/A ratio and e′
Santiago et al. [28]	Depression	Patients with clinically significant depressive symptoms N: 91 Mean age: 58.3 Patients with no depressive symptoms N: 282 Mean age: 56.2 Both sexes	- Patients with clinically significant depressive symptoms had lower septal e′

Abbreviations: A—Late Diastolic Transmitral Flow Velocity; a′—Late Diastolic Mitral Annular Velocity; BD—Bipolar Disorder; BD-I—Bipolar I Disorder; BMI—Body Mass Index; e′—Early Diastolic Mitral Annular Velocity; E/A ratio—Early Diastolic Transmitral Flow Velocity to A Ratio; GLS—Global Longitudinal Strain; HAM-D—Hamilton Depression Rating Scale; IVSD—Interventricular Septum Diameter; LV—Left Ventricular; LVEDD—Left Ventricular End-Diastolic Diameter; LVEF—Left Ventricular Ejection Fraction; LVESD—Left Ventricular End Systole Diameter; LVH—Left Ventricular Hypertrophy; LVM—Left Ventricular Mass; LVMI—Left Ventricular Mass Index; PWD—Posterior Wall Diameter; RWT—Relative Wall Thickness.

**Table 2:** Echocardiographic findings in patients with anxiety disorder, trauma- and stressor-related disorders, anorexia nervosa and sleep disorders.

Study	Psychiatric Disease	Study Group Characteristics	Echocardiographic Findings
Kong et al. [40]	Anxiety Disorder	Patients with hypertension and anxiety disorder N: 56 Age: 45 ± 21 Patients with hypertension only N: 56 Age: 42 ± 23 Both sexes	- Patients with anxiety disorder and hypertension had higher LVMI than patients with hypertension without anxiety disorder
Zhu et al. [41]	Anxiety Disorder	Patients with hypertension and HAM-A score < 14 N: 278 Age: 59.25 ± 9.52 Patients with hypertension and HAM-A score ≥ 14 N: 75 Age: 59.45 ± 11.15 Both sexes	- Patients with anxiety disorder and hypertension had higher LVMI than patients with hypertension without anxiety disorder - The HAM-A score was significantly positively correlated with LVMI
Baykiz et al. [42]	Anxiety Disorder, Stress-Related Disorder	Patients who had recovered from COVID-19 N: 71 Home recovery N: 48 Hospital recovery N: 23 Control group N: 37 Age: 40.4 ± 10 Both sexes	- Patients who suffered from the COVID-19 experienced psychological stress, which caused silent impairment of myocardial contractile function - LA diameter was larger in the hospital recovery group compared with the control group - E/A ratio was significantly lower in the hospital recovery group compared with the home recovery group and the control group - LVEDV, LVESV, LVEDD, LVEF, LAVI, E/e′ ratio, TAPSE, and sPAP were similar in patients and control group - GLS values were significantly less negative in patients than in the control group - GLS significantly negatively correlated with DASS-21 total score, DASS-21 anxiety subscale score, IES-R total score, and IES-R intrusion subscale score - DASS-21 total score was found to be an independent predictor of LV GLS
Oksuz et al. [43]	Anxiety Disorder	Patient group N: 82 Age: 39 ± 8.4 Control group N: 80 Age: 41.6 ± 9 Both sexes	- Both the mean lateral PA interval and mean septal PA interval were significantly prolonged in patients with anxiety disorder compared to a control group - The tricuspid PA interval was similar between the groups - Right intra-AEMD was prolonged in patients with anxiety disorder - Left intra-AEMD was similar between the groups
Kim et al. [44]	Stress-Related Disorder	Patient group GSI ≥ 50 N: 27 Age: 54 ± 8.8 Patient group GSI < 50 Age: 58.3 ± 10.2 Females	- GLS was significantly less negative in patients with GSI ≥ 50 compared to patients with GSI < 50
Hieda et al. [45]	Post-traumatic Stress Disorder	Patient group N: 14 Age: 43.9 ± 11.6 Control group N: 14 Age: 36.3 ± 10.3 Females	- Patients with PTSD had lower mean e′ and Vp and significantly greater E/e′ ratio compared to the control group
Krantz MJ et al. [46]	Anorexia Nervosa	Patient group N: 29 Age: 27.7 ± 7.6 Control group N: 16 Age: 28.5 ± 7.3 Both sexes	- Patients with anorexia nervosa had lower absolute LV internal diastolic dimension and LVM compared to the control group - GLS was similar between patients with anorexia nervosa and the control group - Patients with anorexia nervosa showed less negative basal strain and more negative apical strain compared to the control group - Among patients with anorexia nervosa, longer duration of eating disorder was significantly associated with less negative GLS - In patients with anorexia nervosa, there was a significant interaction between LVM and less negative basal strain
Justine et al. [47]	Anorexia Nervosa	Patient group N: 59 Age: 14.6 ± 1.9 Control group N: 33 Age: 14.1 ± 2.0 Females	- Patients with anorexia nervosa had more negative GLS compared to the control group
Galetta et al. [48]	Anorexia Nervosa	Anorexia Nervosa group N: 25 Age: 17.5 ± 4.2 Constitutionally thin group N: 25 Age: 17.7 ± 3.9 Normal-weight (control) group N: 25 Age: 18.1 ± 4.5 Females	- Patients with anorexia nervosa had reduced LVM with normal systolic function and typical diastolic patterns, characterized by a lower A wave, a comparable E wave and, subsequently, a greater E/A ratio than constitutionally thin and control group
Scheggi et al. [49]	Anorexia Nervosa	Patient group N: 48 Age: 23.7 ± 9.2 Control group N: 44 Age: 24.7 ± 3.6 Both sexes	- GLS was significantly less negative in patients with anorexia nervosa compared to the control group
Borgia et al. [50]	Anorexia Nervosa	Patient group N: 38 Age: 15.6 ± 2.3 Control group N: 40 Age: 15.7 ± 1.8 Both sexes	- Anorexia nervosa cardiopathy was associated with significantly less negative GLS, reduced LVEDD and LVM, higher E wave, E/A and E/e′ ratios, and LV sphericity index values compared to the control group and anorexia nervosa children without cardiopathy
Hanachi et al. [51]	Anorexia Nervosa	Patient group N: 124 Age: 32 ± 2.8 Control group N: 29 Age: 32 ± 10 Both sexes	- LV and RV myocardial systolic and diastolic velocities were significantly reduced in patients with anorexia nervosa
Galetta et al. [52]	Anorexia Nervosa	Anorexia group N: 20 Age: 22.4 ± 4.3 Thinness group N: 20 Age: 23.2 ± 3.8 Control group N: 20 Age: 23.5 ± 4.1 Females	- Patients with anorexia nervosa had lower LVM and s′ peak of both lateral wall and septum, and comparable e′, a′, and e′/a′ ratio - The ratio E/e′ was significantly greater in anorexic patients than in controls - In patients with anorexia nervosa s′ was significantly related to LVMI, at both septum and lateral wall levels
Strand et al. [53]	Insomnia	Patient group N: 410 Control group N: 378 Both sexes	- There was no evidence of an association between increasing numbers of insomnia symptoms and any outcome variable related to LV function
Gump et al. [54]	Sleep Disorder	Patient group N: 176 Age: 9–11 Both sexes	- Poor sleep quality was associated with significantly greater LVM, RWT, and RV dimension
Lee et al. [55]	Sleep Disorder	31,598 healthy adults Age: 39.2 ± 7.0 Both sexes	- Short sleep duration was associated with greater LVH and increased RWT - Among individuals with normal sleep duration, poor quality of sleep was not associated with adverse cardiac measures

Abbreviations: a′—Late Diastolic Mitral Annular Velocity; AEMD—Atrial Electromechanical Delay; DASS-21—Depression, Anxiety, and Stress Scale-2; E/A ratio—Ratio of the Early Diastolic Transmitral Flow Velocity (E) to the Late Diastolic Transmitral Flow Velocity (A); E/e′ ratio—Ratio of E to the Early Diastolic Mitral Annular Velocity; GLS—Global Longitudinal Strain; GSI—Global Stress Severity Index; HAM-A—Hamilton Anxiety Rating Scale; IES-R—Impact of Events Scale; LA—Left Atrial; LAVI—Left Atrial Volume Index; LV—Left Ventricular; LVEDD—Left Ventricular End-Diastolic Diameter; LVEDV—Left Ventricular End-Diastolic Volume; LVEF—Left Ventricular Ejection Fraction; LVESV—Left Ventricular End-Systolic Volume; LVH—Left Ventricular Hypertrophy; LVM—Left Ventricular Mass; LVMI—Left Ventricular Mass Index; PA—Time Interval From The Onset Of P-Wave On Surface Electrocardiogram To The Beginning Of A-Wave Interval With Tissue Doppler Echocardiography; PTSD—Post-Traumatic Stress Disorder; RWT—Relative Wall Thickness; RV—Right Ventricular; s′—Myocardial Systolic Wave; sPAP—Pulmonary Artery Systolic Pressure; TAPSE—Tricuspid Annular Plane Systolic Excursion; Vp—Velocity of Propagation.

**Table 3:** Echocardiographic findings in patients with substance-related and addictive disorders, neurocognitive disorders and borderline personality disorder.

Study	Psychiatric Disease	Study Group Characteristics	Echocardiographic Findings
Kucuk et al. [75]	Substance Use Disorder	Patient group N: 41 Age: 43.17 ± 1.4 Control group N: 30 Age: 38.0 ± 1.73 Males	- LV GLS and GCS were significantly less negative in alcoholic patients compared with the control group
Wang et al. [76]	Substance Use Disorder	Asymptomatic alcoholic patients in three groups of increasing alcohol intake (mild, moderate and severe) N: 92 Age: 55 ± 8 Control group N: 30 Age: 54 ± 7 Males	- The moderate and severe alcohol intake groups demonstrated less negative GLS and GCS, less positive GRS values, and reduced E/A ratios compared with the mild alcohol intake group and the control group
Kocabay et al. [77]	Substance Use Disorder	Asymptomatic alcoholic patients N: 75 Age: 39.8 ± 6.5 Control group N: 30 Age: 34.8 ± 5.8 Males	- Alcoholic patients had longer deceleration time, lower E/A and Vp, and higher E/e′ - LA function was reduced in alcoholic patients
Xu et al. [78]	Substance Use Disorder	Asymptomatic alcoholic patients in three groups of increasing alcohol intake (mild, moderate, severe) N: 92 Control group N: 30 Males	- Alcoholic patients had larger LA volumes - GLS was less negative in moderate and severe drinking groups, compared with mild drinking and control group - E/A ratio showed a progressive decrease according to the grades of alcoholism
Meng et al. [79]	Substance Use Disorder	Asymptomatic alcoholic patients in three groups (A, B, C) of increasing alcohol intake N: 92 Group A: ≥90 mg ethanol or ≥2–3 bottles of beer, 3–5 days/week for 5–8 years Group B: ≥90 mg ethanol or ≥2–3 bottles of beer, 3–5 days/week for 9–20 years Group C: ≥150 mg ethanol or ≥4 bottles of beer, 6–7 days/week for >10 years Age: 42–65 Control group N: 30 Age: 40–65 Males	- Patients with the highest alcohol consumption had significantly higher RVEDD and significantly lower TAPSE compared to patients with lower alcohol consumption and the control group. In these patients, all the diastolic and systolic parameters P, S, and SRs were significantly lower compared to the other groups - In the group with medium alcohol consumption, the longitudinal SRe and SRa of the RV free wall were significantly lower and the LV longitudinal strain was less negative than in the group with lower alcohol consumption or in the control group - A significant negative linear correlation was noted between global RV systolic parameters—S, SRs, and TAPSE
Zhang et al. [80]	Substance Use Disorder	Methamphetamine Abusers N: 53 Age: 16–32 Control group N: 53 Age: 17–31 Males	- GAS, GLS, and GCS were significantly less negative in methamphetamine abusers compared to the control group
Demirkıran et al. [81]	Substance Use Disorder	Heroin users N: 31 Age: 24 ± 4 Synthetic cannabinoid users N: 30 Age: 24 ± 6 Control group N: 32 Age: 25 ± 8 Males	- There were no differences in baseline characteristics or 2D echocardiographic values between the heroin users, SCB users and the control group - SCB may cause subclinical LV dysfunction
Sanna et al. [82]	Alzheimer’s Disease	Patient group N: 32 Age: 75.1 ± 7.2 Control group N: 34 Age: 73.3 ± 6.2 Both sexes	- Patients with AD had greater MWT and IVS, significantly lower E/A ratio and a trend toward a higher E/e′ ratio compared to the control group
Nowak et al. [83]	Dementia	Patients with SDD N: 42 Age: 69–81 Patients without SDD N: 97 Age: 66–77 Both sexes	- RWT was moderately associated with SDD in patients following HF decompensation
Hashemi et al. [84]	Mild Cognitive Impairment	Patient group N: 85 Age: 65.78 ± 5.13 Both sexes	- The lower LVEF correlated with lower scores on visuospatial, executive function, processing speed/attention and verbal memory capacities - A higher LA size correlated with lower executive function and processing speed/attention - In the group of patients with an impaired cognitive state, the e′ index was associated with lower capacity of executive function
Çalık et al. [85]	Alzheimer’s Disease	Patient group N: 29 Age: 76.8 ± 5.2 Control group N: 24 Age: 77.1 ± 4.9 Both sexes	- E wave was lower, and A wave, deceleration time of peak E velocity, and IVRT were higher in patients with AD - e′ was significantly lower in AD patients, whereas a′ and E/e′ ratio were similar between AD and the control group
Engemann et al. [86]	Borderline Personality Disorder	Patient group N: 50 Control group N: 50 Females	- Patients with BPD had less negative GLS compared to the control group - Allostatic load was significantly associated with GLS, with childhood trauma approaching significance
Aweimer et al. [87]	Borderline Personality Disorder	Patient group N: 50 Age: 23.85 ± 5.2 Control group N: 50 Age: 24.10 ± 3.9 Females	- Patients with BPD had significantly thicker IVS and lower TAPSE compared to the control group - GLS was significantly less negative in patients with BPD - The number of pathological segments of the LV was higher in patients with BPD compared to the control group

Abbreviations: A—Late Diastolic Transmitral Flow Velocity; a′—Late Diastolic Mitral Annular Velocity; AD—Alzheimer’s Disease; BPD—Borderline Personality Disorder; E—Early Diastolic Transmitral Flow Velocity; e′—Early Diastolic Mitral Annular Velocity; GAS—Global Area Strain; GCS—Global Circumferential Strain; GLS—Global Longitudinal Strain; GRS—Global Radial Strain; HF—Heart Failure; IVRT—Isovolumic Relaxation Time; IVS—Interventricular Septum; LA—Left Atrial; LV—Left Ventricular; LVEF—Left Ventricular Ejection Fraction; MWT—Maximum Wall Thickness; P—Peak Systolic Displacement; RWT—Relative Wall Thickness; RV—Right Ventricular; RVEDD—Right Ventricular End-Diastolic Dimension; S—Systolic Strain Peak; SCB—Synthetic Cannabinoids; SDD—Screening Diagnosis of Dementia; SRa—Late Diastolic Strain Rate; SRe—Early Diastolic Strain Rate; SRs—Systolic Strain Rate; TAPSE—Tricuspid Annular Plane Systolic Excursion; Vp—Velocity of Propagation.

## Data Availability

No new data were created.
